# Better Prognosis of Gastric Neuroendocrine Carcinoma Than Gastric Adenocarcinoma among Whites in the United States: A Propensity Score Matching Analysis Based on SEER

**DOI:** 10.3390/curroncol29070387

**Published:** 2022-07-11

**Authors:** Zefeng Li, Hu Ren, Lulu Zhao, Xiaojie Zhang, Tongbo Wang, Chongyuan Sun, Penghui Niu, Wanqing Wang, He Fei, Chunguang Guo, Yingtai Chen, Dongbing Zhao

**Affiliations:** Department of Pancreatic and Gastric Surgical Oncology, National Cancer Center/National Clinical Research Center for Cancer/Cancer Hospital, Chinese Academy of Medical Sciences and Peking Union Medical College, Beijing 100021, China; lizefeng1997@126.com (Z.L.); renhu0001@163.com (H.R.); lulu_2019@163.com (L.Z.); xiaojiezhang0925@163.com (X.Z.); wangtb9212@163.com (T.W.); sunchongyuan@126.com (C.S.); m17853137288@163.com (P.N.); wangwq2021@126.com (W.W.); feihe1997@163.com (H.F.); chunguang_guo@outlook.com (C.G.)

**Keywords:** gastric cancer, gastric adenocarcinoma, gastric neuroendocrine carcinoma, prognosis, survival outcome

## Abstract

It was generally believed that the prognosis of gastric neuroendocrine carcinoma (GNEC) was worse than gastric adenocarcinoma (GAC). However, almost all previous studies compared the prognosis of GNEC and GAC based on East Asians. In this study, we evaluated the clinicopathological features and prognosis of GNEC and GAC in Whites. Patients with GNEC and GAC were identified from 2000 to 2018 in the Surveillance, Epidemiology, and End Results (SEER) database. We used propensity score matching (PSM) analysis to match the age, sex, TNM stage, and treatments received between GNEC and GAC, then compared the overall survival (OS) and cancer-specific survival (CSS) in the two types. A total of 392 cases of GNEC and 12,835 cases of GAC in Whites were recognized. After PSM, the 5-year OS rates of GNEC and GAC were 50.3% and 43.0%, respectively (*p* = 0.010). The 5-year CSS rates of GNEC and GAC were 57.4% and 50.1%, respectively (*p* = 0.012). Besides, multivariable cox regression analyses showed that GNEC was an independent predictor of improved OS (HR 0.719; 95% CI 0.607–0.853) and CSS (HR 0.691; 95% CI 0.571–0.835) in the matched data. The prognosis of GNEC was better than GAC in Whites, showing significant ethnic differences. Appropriate treatments and follow-up strategies for GNEC in Whites are probably different from East Asians. The potential genetic and molecular mechanisms need to be further explored.

## 1. Introduction

Gastric cancer is the fifth most common malignant tumor all over the world with the fourth highest mortality [1]. Among all pathological types of gastric cancer, adenocarcinoma is the most common type [2]. However, compared with the decreased incidence rate of gastric adenocarcinoma (GAC), the incidence rate of gastric neuroendocrine carcinoma (GNEC) has increased rapidly in recent years [3]. In 2019, WHO classified gastric neuroendocrine neoplasms into three types: gastric neuroendocrine tumors, gastric neuroendocrine carcinoma, and mixed neuroendocrine/non-neuroendocrine neoplasms [4]. Given that the biological behavior and prognosis of GNEC are more like GAC rather than gastric neuroendocrine tumors, the stage and treatments for GNEC mainly refer to GAC [5,6,7].

It was generally believed that the prognosis of GNEC was worse than GAC [8,9,10,11,12,13,14,15,16,17]. In 2006, Japanese doctor Jiang et al. [16] found that the 5-year overall survival (OS) rates for the GNEC and GAC were 31.1% and 69.3%, and the OS was very significantly different between GNEC and GAC within each stage. Recently, Changming Huang et al. [17] conducted a multicenter clinical retrospective study comparing the prognosis of 503 cases of GNEC and 2785 cases of GAC from 23 Chinese hospitals. After propensity matching analysis (PSM), 5-year disease-free survival was 47.6% for patients with GNEC and 57.6% for patients with GAC (*p* < 0.001). Although the threshold was controversial, containing neuroendocrine components was regarded as a symbol of poor prognosis in gastric cancer [10,18]. Therefore, for GNEC, more aggressive treatments were usually used, such as enlarging the resection range [19]. However, almost all the previous studies focused on GNEC and GAC in East Asians. Furthermore, lots of research studies have previously revealed the marked survival disparities of GAC among different races, as they have in neuroendocrine neoplasms [20,21,22]. Hence, whether the prognosis of GNEC is worse than GAC in other racial patients remains unknown. There is a need to compare the prognosis of GNEC and GAC in other races, the results of which are important to design follow-up and treatment strategies for patients with GNEC.

As such, in this study, we evaluated the clinicopathological features and prognosis of GNEC in comparison to GAC in Whites. To our knowledge, this is the first research comparing prognosis between GNEC and GAC in Whites.

## 2. Materials and Methods

### 2.1. SEER Database and Patients Selection

The Surveillance, Epidemiology, and End Results (SEER) Database (www.seer.cancer.gov) is a national database that comprises 28% of the US population. We used “SEER Research Plus Data, 18 Registries, Nov 2020 Sub (2000–2018)” to retrieve demographic or clinicopathological data of patients with GNEC and GAC. The database was released in April 2021 and based on the November 2020 submission. We identified GAC patients with the primary site code as “C16.0–C16.9, stomach” and the following International Classification of Diseases for Oncology, Third Edition (ICD-O-3) histology codes: “8140 (Adenocarcinoma, NOS), 8143 (Superficial spreading adenocarcinoma), 8144 (Adenocarcinoma, intestinal type), 8210 (Adenocarcinoma in adenomatous polyp), 8211 (Tubular adenocarcinoma), 8255 (Adenocarcinoma with mixed subtypes), 8260 (Papillary adenocarcinoma, NOS), 8261 (Adenocarcinoma in villous adenoma), 8262 (Villous adenocarcinoma), 8263 (Adenocarcinoma in tubulovillous adenoma), 8310 (Clear cell adenocarcinoma, NOS), 8323 (Mixed cell adenocarcinoma), 8441 (Serous cystadenocarcinoma, NOS), 8480 (Mucinous adenocarcinoma), 8481 (Mucin-producing adenocarcinoma), 8570 (Adenocarcinoma with squamous metaplasia), 8574 (Adenocarcinoma with neuroendocrine differentiation), and 8576 (Hepatoid adenocarcinoma) ”. We also identified GNEC patients with the primary site code as “C16.0–C16.9, stomach” and the following ICD-O-3 histology codes: “8013 (Large cell neuroendocrine carcinoma), 8041 (Small cell carcinoma, NOS), and 8246 (Neuroendocrine carcinoma, NOS)”. The exclusion criteria were as follows: cases with other malignancies, cases without follow-up information, cases with unknown treatment details, cases with unknown race, cases with unknown T/N/M stage, and cases with unknown tumor size. Finally, 392 White patients with GNEC and 12835 White patients with GAC were recognized (Supplementary Figure S1). The data in this study were obtained from the SEER database under the SEER data use agreement (ID: 17851-Nov2020).

### 2.2. Data Analysis 

Demographic or clinicopathological data were collected, including age, sex, race, tumor site, tumor size, T stage, N stage, M stage, and treatments received. According to “Race/ethnic”, races of cases were grouped into Whites (including White Hispanic and Latino Americans), East Asians (Chinese, Japanese, and Korean), and other races. In the light of “Primary Site—labeled”, we divided the patients into five categories, including proximal (cardia, fundus), middle (body, lesser curvature, greater curvature of stomach), distal stomach (antrum, pyloric), mix (overlapping), and unknown (stomach, NOS), according to the anatomical location. TNM stage was reevaluated according to the 8th AJCC staging definition for gastric cancer. We defined stage IA as the early stage, stage IB-III as the locally advanced stage, and stage IV as the distant metastatic stage to further analyze. OS and cancer-specific survival (CSS) rates were calculated from the time of diagnosis to the date of death or the date of last follow-up. OS, CSS, and other causes of death were determined from SEER cause of death data. 

### 2.3. Statistical Analysis 

PSM analysis was used to adjust for the imbalance between GNEC and GAC. Age, sex, TNM stage, receiving surgery or not, and treatment received were included in the logistic regression to predict the propensity score. Within a caliper of 0.01, the nearest-neighbor method was used to perform a 1–3 matching procedure without replacement. PSM was performed using R version 4.0.4 (R Project for Statistical Computing).

Continuous variables were non-normally distributed and represented by median (25th–75th quartile), and compared using the Mann–Whitney U test. Categorical variables were represented by numbers and percentages and compared using x^2^ test or Fisher exact test appropriately. Kaplan–Meier survival analyses were conducted to estimate median survival time and survival rates. Additionally, the log-rank test was used to compare OS and CSS between GNEC and GAC. Univariable Cox regression was performed and the variables with *p* < 0.1 were included in the multivariable Cox model to determine the independent prognostic factors associated with OS and CSS. Statistical analyses were completed in SPSS statistical software version 26.0 (IBM, New York, NY, USA) with a critical level of significance of *p* < 0.05.

## 3. Results

### 3.1. Baseline Characteristics and Survival Outcomes between GNEC and GAC in Whites before PSM

**Figure 1:**
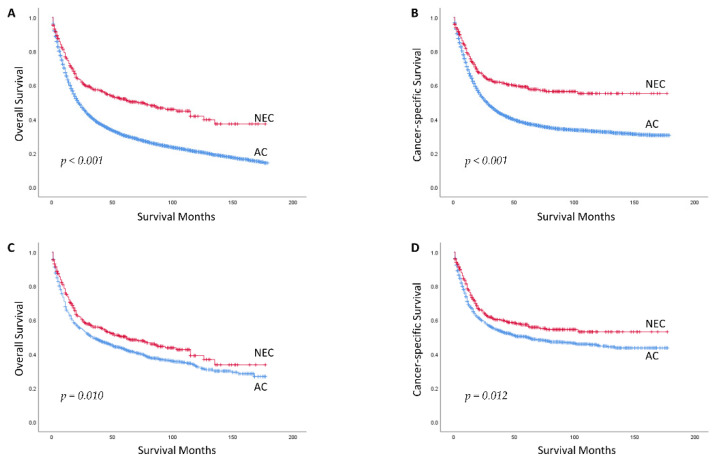
Kaplan–Meier Survival Curves for White Patients with Gastric Neuroendocrine Carcinoma and Adenocarcinoma before ((**A**): OS; (**B**): CSS) and after PSM ((**C**): OS; (**D**): CSS). NEC: Neuroendocrine Carcinoma; AC: Adenocarcinoma; PSM: Propensity Score Matching; OS: Overall Survival; CSS: Cancer-specific Survival.

A total of 392 GNEC cases and 12,835 GAC cases were finally identified (Table 1). The ages of GNEC patients were younger than GAC (median [25th–75th quartile], 63.50 years [51–71.75] vs. 68 years [59–77]). Compared to GAC patients, GNEC patients were more prone to show a balance between male (51.8%) and female (48.2%), have more tumors located in the middle of stomach (35.2% vs. 19.2%), and have smaller tumors (median [25th–75th quartile], 2.5 cm [1–5.5] vs. 4.0 cm [2.5–6.0]). Besides, the proportion of T1 or T2 in GNEC was higher than GAC (37.5% or 24.5% vs. 26.4% or 12.7%), and the proportion of N0 in GNEC was higher than GAC (61.0% vs. 41.1%). However, GNEC patients were more likely to have distant metastasis at the time of diagnosis (25.8% versus 19.2%). There was no significant difference in the proportion of patients undergoing surgery between GNEC and GAC. However, in terms of receiving chemotherapy or/and radiotherapy, the proportion of patients with GNEC was lower than GAC (31.6% vs. 60.3%). There were statistically significant differences in age, sex, tumor location, tumor size, T/N/M stage, TNM stage, and receiving chemotherapy or/and radiotherapy proportion between GNEC and GAC. As Kaplan–Meier survival curves showed (Figure 1A,B), no matter OS or CSS, patients with GNEC had a better prognosis than GAC (both *p* < 0.001). 

### 3.2. Baseline Characteristics and Survival Outcomes between GNEC and GAC in Whites after PSM

After PSM, 371 patients with GNEC and 1059 patients with GAC were included in the matched data set. There were no more significant differences in age, sex, TNM stage, and treatments received between patients with GNEC and those with GAC (Table 2). 

The median (range) follow-up time was 69 (1–177) months for patients with GNEC and 91 months (1–177) for patients with GAC in the matched data. The median OS for GNEC and GAC patients was 62 and 34 months, respectively. The median CSS for GAC was 61 months, and the median CSS for GNEC did not reach (Table 2). As the Kaplan–Meier survival curves showed (Figure 1C,D), no matter OS (*p* = 0.010) or CSS (*p* = 0.012), patients with GNEC still had a better prognosis than GAC. For patients with GNEC, the 1-year, 3-year, and 5-year OS and CSS were 75.1%, 56.0%, and 50.3% and 77.7%, 60.3%, and 57.4%, respectively. For patients with GAC, the 1-year, 3-year, and 5-year OS and CSS were 65.9%, 49.1%, and 43.0% and 69.2%, 54.2%, and 50.1%, respectively (Table 2). In the multivariable regression analysis, GNEC was validated as an independent predictor of better OS (HR 0.719, 95% CI [0.607, 0.853]) and CSS (HR 0.691, 95% CI [0.571, 0.835]), compared to GAC (Table 3 and Table 4). 

### 3.3. Subgroup Analysis According to Stage in Whites

To further compare the prognosis of GNEC with GAC in Whites, we divided gastric cancer patients into the early stage group, the locally advanced stage group, and the distant metastatic stage group. Then, we compared the prognosis of GNEC and GAC in different subgroups. The basis characteristics before PSM are shown in Supplementary Tables S1, S5, and S9. After PSM, there were no statistically significant differences in age, sex, and treatments received between patients with GNEC and GAC in the three subtypes (Supplementary Tables S2, S6, and S10). In the early stage group, there were 96 cases of GNEC and 263 cases of GAC included in the matched data. In the locally advanced stage group, 159 cases of GNEC and 428 cases of GAC were matched. In the distant metastatic stage group, 99 cases of GNEC and 277 cases of GAC were included in the matched data.

**Figure 2:**
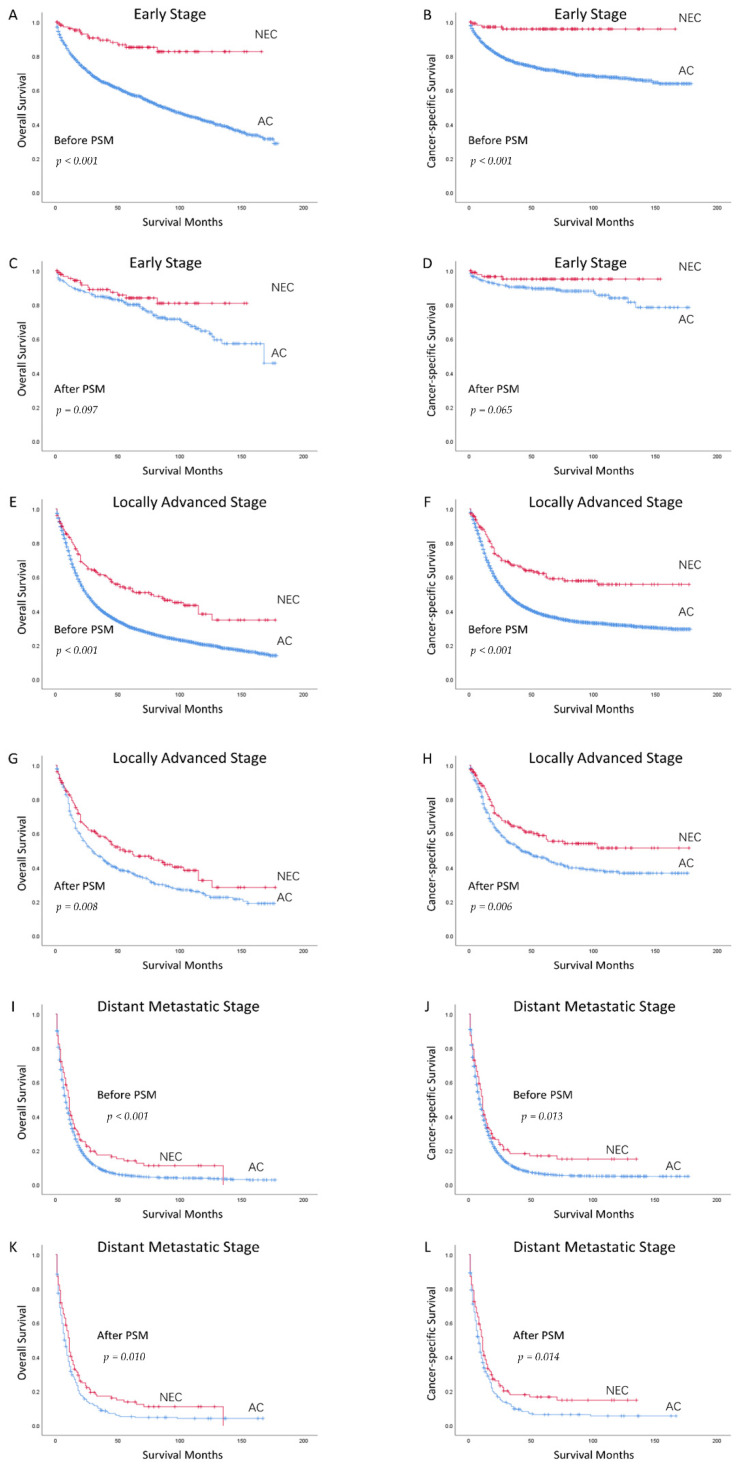
Kaplan–Meier Survival Curves for White Patients with Different Stage Gastric Neuroendocrine Carcinoma and Adenocarcinoma before and after PSM (Early Stage before PSM: (**A**): OS, (**B**): CSS; Early Stage after PSM: (**C**): OS, (**D**): CSS; Locally Advanced Stage before PSM: (**E**): OS, (**F**): CSS; Locally Advanced Stage after PSM: (**G**): OS, (**H**): CSS; Distant Metastatic Stage before PSM: (**I**): OS, (**J**): CSS; Distant Metastatic Stage after PSM: (**K**): OS, (**L**): CSS). NEC: Neuroendocrine Carcinoma; AC: Adenocarcinoma; PSM: Propensity Score Matching; OS: Overall Survival; CSS: Cancer-specific Survival.

In the early stage patients, as Kaplan–Meier survival curves showed (Figure 2A,B), the OS and CSS of patients with GNEC were better than those with GAC before PSM (both *p* < 0.001). After PSM (Figure 2C,D), the prognosis of GNEC was also better than GAC, but with no statically significant difference (OS *p* = 0.097, CSS *p* = 0.065). In the multivariable analysis (Supplementary Tables S3 and S4), GNEC was recognized as an independent prognostic factor associated with better outcomes (OS HR 0.528, 95% CI [0.280, 0.996]; CSS HR 0.319, 95% CI [0.105, 0.970]).

In the locally advanced stage patients, as Kaplan–Meier survival curves showed (Figure 2E,F), the OS and CSS of patients with GNEC were better than those with GAC before PSM (both *p* < 0.001). Besides, after PSM (Figure 2G,H), the OS and CSS of patients with GNEC were still better than patients with GAC (OS *p* = 0.008, CSS *p* = 0.006). Although GNEC was not identified as an independent prognostic factor for OS in the multivariable analysis (HR 0.803, 95% CI [0.621, 1.038]) (Supplementary Table S7), GNEC was an independent prognostic factor related to better CSS (HR 0.725, 95% CI [0.535, 0.981]) (Supplementary Table S8).

In the distant metastatic stage patients, as the Kaplan–Meier survival curves showed (Figure 2I,J), the OS and CSS of patients with GNEC were better than those with GAC before PSM (OS *p* < 0.001, CSS *p* = 0.013). In addition, after PSM (Figure 2K,L), there was still a statistically significant difference between the two types (OS *p* = 0.010, CSS *p* = 0.014). Furthermore, GNEC was an independent prognostic factor for the distant metastatic stage gastric cancer in the multivariable analysis (OS HR 0.655, 95% CI [0.508, 0.843]; CSS HR 0.649, 95% CI [0.501, 0.840]) (Supplementary Tables S11 and S12).

### 3.4. Baseline Characteristics and Survival Outcomes between GNEC and GAC in East Asians

**Figure 3:**
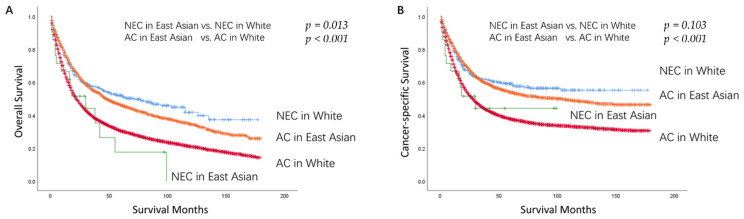
Kaplan–Meier Survival Curves for GNEC and GAC in different races in the Unmatched Data ((**A**): OS; (**B**): CSS). NEC: Neuroendocrine Carcinoma; AC: Adenocarcinoma; OS: Overall Survival; CSS: Cancer-specific Survival.

The baseline characteristics of patients with GNEC and GAC in East Asians are shown in Supplementary Tables S13 and S14. Before PSM, there was an imbalance between treatments received in different groups, and after PSM, the imbalance was eliminated. To verify the conclusions of previous studies, we compared the prognosis of East Asian patients with GNEC and GAC (Supplementary Figure S2). Although with small sample size, the OS of GAC seemed to be better than GNEC. The *p* value was 0.019 in the unmatched data and the *p* value was 0.052 in the matched data. In terms of CSS, there was no statistically significant difference in prognosis between the two groups. The *p* value was 0.104 in the unmatched data and 0.229 in the matched data. Besides, we showed the survival curves of GNEC and GAC among different races in the unmatched data (Figure 3). The prognosis of GNEC in Whites seemed to be better than in East Asians (OS *p* = 0.013, CSS *p* = 0.103). The prognosis of GAC in East Asians was better than in Whites (OS, CSS both *p* < 0.001).

## 4. Discussion

In this study, we evaluated the clinicopathological features and prognosis of GNEC in comparison to GAC in Whites. To our knowledge, this study is the first to compare the prognosis of GNEC and GAC in Whites. Inconsistent with the results of previous research studies focused on East Asians [8,9,10,11,12,13,14,15,16,17], we found that the prognosis of GNEC was better than GAC in Whites. 

It was believed that the prognosis of GNEC was worse than that of GAC. Containing neuroendocrine components was regarded as an indicator of poor prognosis in GAC. In 2013, Ishida et al. [15] compared the stage-specific 5-year overall survival rates of the 51 patients with GNEC and 1035 cases with GAC who were surgically treated in Japan. The survival rates of the patients with GNEC were poorer. In 2014, Korean doctor Park et al. [14] suggested that the survival rates of patients with >10% neuroendocrine differentiation were significantly poorer than those with <10% neuroendocrine differentiation. Furthermore, Kim et al. [12], comparing relapse-free survival of 63 cases of GNEC and 762 cases of GAC, concluded that non-advanced GNEC showed poorer relapse-free survival than GAC. A previous study of our center also came to a similar conclusion [18]. GNEC was regarded as a more malignant pathological type than GAC. Therefore, to improve the survival time of patients with GNEC, more active and effective multidisciplinary treatments and additional close follow-up strategies were undertaken for patients with GNEC [17]. However, all of the previous studies were based on East Asians.

We distinguished 392 cases of GNEC and 12,835 cases of GAC in Whites from the SEER database. Considering the imbalance between the characteristics baseline of the two groups, we performed the PSM. After PSM, factors that may affect prognosis were balanced between two groups, including age, sex, stage, and treatments received. Contrary to the previous studies, we found that patients with GNEC had a better prognosis than GAC in Whites, which was revolutionary. (Figure 1C,D), no matter the OS (*p* = 0.010) or CSS (*p* = 0.012). On top of that, GNEC remained an independent prognostic factor by multivariable analysis (Table 3 and Table 4). Besides, we further divided the cohort into the early stage, the locally advanced stage, and the distant metastatic stage subgroups. GNEC showed a trend of better prognosis than GAC in all subgroups. Our findings were quite different from previous studies [8,9,10,11,12,13,14,15,16,17]. The most likely reason is that the samples we chose were different from the previous research studies. The outcome illustrated that there is a disparity in the comparison of prognosis between GNEC and GAC among different races.

The second finding in this study was that race might be an important prognostic factor for patients with GNEC (Figure 3). In the unmatched data of the present study, we showed the survival curves of GNEC and GAC among different races, and the prognosis of GNEC in Whites seemed to be better than in East Asians (OS *p* = 0.013). However, there is no statically significant difference for CSS in the two groups (CSS *p* = 0.103). The reason might be the small number of East Asian patients with GNEC, and this conclusion needs further validation. Dasari et al. [21] found that in distant gastrointestinal neuroendocrine neoplasms, Asians or Pacific Islanders were identified as an independent risk factor associated with poor prognosis compared with Whites. However, a recent study [23] did not recognize race as an independent risk factor related to prognosis in patients with GNEC. In this study, with 653 GNEC patients, there were just three categories of the race including Whites, Blacks, and others, which did not split up Asians separately as a race. Furthermore, we confirmed the previous studies’ conclusions. Firstly, the prognosis of GNEC was worse than GAC in East Asians (Supplementary Figure S2) [8,9,10,11,12,13,14,15]. Secondly, the prognosis of GAC in East Asians was better than those in Whites (Figure 3) [24,25,26].

Besides, we found that most of the clinicopathological features of GNEC in Whites were similar to the previous study except for sex. In our review of 392 patients with GNEC, the median age of the patients was 63.5 years, which was similar to the previous study [17]. GNEC was reported to always be seen in the upper third of the stomach just as reported [8,15]. Compared with GAC, the GNEC seemed to be more frequent distant metastasis at diagnosis, corresponding to what Kubota once reported [13]. Differently, Ishida [15] and Huang [17] reported that GNEC was more common in males than in females, with a ratio of approximately 3:1. However, in our research, the ratio between males and females was nearly 1:1, showing a racial difference.

Our study has numerous strengths. Firstly, a large population database was utilized to compare the prognosis of GNEC and GAC in Whites, and a relatively great quantity of patients with GNEC, an “orphan” disease, were identified, leading to an adequately powered study. As far as I know, this is the first study focused on Whites, because all of the relevant previous research studies have been based on East Asians. Secondly, PSM was conducted to eliminate the observed bias in baseline covariates between the GNEC and GAC groups. We further confirmed our conclusion in different stages of GNEC. Lastly, we validated the previous conclusion that the prognosis of GNEC was worse than GAC in East Asians and the prognosis of GAC was worse in Whites than in East Asians. That could prove the reliability of our data and conclusions. There were also some limitations to our study. Firstly, specific pathological information and treatment details, such as the expression of Ki67 and chemotherapy regimen, were unavailable in the SEER database, impeding the opportunity to further eliminate the imbalance between GNEC and GAC. Moreover, because of the lack of information in several cases, we had to give up some patients, resulting in a further reduction of samples. Besides, the GAC group consists of a heterogeneous group of subtypes. The prognostic curves on GAC may not be representative for all of the subtypes within the group. Finally, socioeconomic- or treating facility-related factors were not included. Nevertheless, in consideration of the rarity of GNEC, our study has included a relatively large sample size, which could suggest the need for paying attention to racial disparities in the prognosis of GNEC.

The results and conclusions of the present study were very meaningful. Treatment and follow-up patterns are determined according to the disease’s prognosis. In the past, GNEC was regarded as a malignant pathological type and treated with more radical treatment, such as salvage surgery after endoscopic resection for early GNEC, additional close follow-up strategies, and more aggressive adjuvant therapies [17]. However, in our study, we found that the prognosis of GNEC was better than that of GAC, which could influence the treatment decisions and follow-up period in Whites. Better understanding the prognosis of GNEC will not only help reduce the medical cost burden, but also the patients’ psychological burden. Besides, we found that there is a potential prognosis disparity of GNEC among different races. The exploration of molecular mechanism may help to find potential therapeutic targets that will help to abolish these disparities in the future.

## 5. Conclusions

We first discovered that the prognosis of GNEC was better than GAC in Whites. This will help us to further understand GNEC, especially its racial difference. A better understanding could lead to more appropriate treatment patterns and reduce unnecessary medical costs and psychological burden. The treatments and follow-up duration of GNEC might be adjusted according to different races. Further validation and potential genetic and molecular mechanisms among different racial groups should be investigated in the future, so as to further improve the prognosis of patients with GNEC.

## Figures and Tables

**Table 1:** Baseline Clinicopathologic Characteristics of White Patients with Gastric Neuroendocrine Carcinoma and Adenocarcinoma in the Unmatched Data.

	No. (%)	No. (%)	*p*
	**NEC (392)**	**AC (12,835)**	
**Characteristic**			
**Age, median (IQR)**	63.50 (51–71.75)	68 (59–77)	<0.001
**Sex**			<0.001
Men	203 (51.8)	9052 (70.5)	
Women	189 (48.2)	3783 (29.5)	
**Tumor location**			<0.001
Proximal	123 (31.4)	6802 (53.0)	
Middle	138 (35.2)	2459 (19.2)	
Distal	52 (13.3)	2141 (16.7)	
Mix	18 (4.6)	722 (5.6)	
Unknown	61 (15.5)	711 (5.5)	
**Tumor size, median (IQR), cm**	2.5 (1–5.5)	4.0 (2.5–6.0)	<0.001
**T stage**			<0.001
1	147 (37.5)	3392 (26.4)	
2	96 (24.5)	1626 (12.7)	
3	82 (20.9)	4858 (37.8)	
4a	32 (8.2)	1823 (14.2)	
4b	35 (8.9)	1136 (8.9)	
**N stage**			<0.001
0	239 (61.0)	5281 (41.1)	
1	105 (26.8)	4028 (31.4)	
2	28 (7.1)	1908 (14.9)	
3a	16 (4.1)	1250 (9.7)	
3b	4 (1.0)	368 (2.9)	
**M stage**			<0.001
0	291 (74.2)	10,377 (80.8)	
1	101 (25.8)	2458 (19.2)	
**TNM stage**			<0.001
I	183 (46.7)	3398 (26.5)	
II	68 (17.3)	3540 (27.6)	
III	40 (10.2)	3439 (26.8)	
IV	101 (25.8)	2458 (19.1)	
**Surgery**			0.364
No	118 (30.1)	3595 (28.0)	
Yes	274 (69.9)	9240 (72.0)	
**Receiving chemotherapy or/and radiotherapy**			<0.001
No	268 (68.4)	5097 (39.7)	
Yes	124 (31.6)	7738 (60.3)	

NEC: Neuroendocrine Carcinoma; AC: Adenocarcinoma; OS: Overall Survival; CSS: Cancer-specific Survival; IQR: Interquartile Range.

**Table 2:** Baseline Clinicopathologic Characteristics and Survival Outcomes of White Patients with Gastric Neuroendocrine Carcinoma and Adenocarcinoma in the Matched Data.

	No. (%)	No. (%)	*p*
	**NEC (371)**	**AC (1059)**	
**Characteristic**			
**Age, median (IQR)**	65 (54–72)	66 (55–73)	0.182
**Sex**			0.429
Men	200 (53.9)	596 (56.3)	
Women	171 (46.1)	463 (43.7)	
**Tumor location**			<0.001
Proximal	116 (31.3)	574 (54.2)	
Middle	129 (34.8)	206 (19.5)	
Distal	50 (13.5)	175 (16.5)	
Mix	18 (4.9)	45 (4.2)	
Unknown	58 (15.6)	59 (5.6)	
**Tumor size, median (IQR), cm**	2.5 (1–6.0)	2.0 (1.0–4.0)	0.012
**T stage**			<0.001
1	133 (35.8)	517 (48.8)	
2	92 (24.8)	109 (10.3)	
3	80 (21.6)	255 (24.1)	
4a	31 (8.4)	104 (9.8)	
4b	35 (9.4)	74 (7.0)	
**N stage**			0.207
0	220 (59.3)	626 (59.1)	
1	103 (27.8)	248 (23.4)	
2	28 (7.5)	112 (10.6)	
3a	16 (4.3)	62 (5.9)	
3b	4 (1.1)	11 (1.0)	
**M stage**			0.405
0	271 (73.0)	798 (75.4)	
1	100 (27.0)	261 (24.6)	
**TNM stage**			0.422
I	165 (44.5)	470 (44.4)	
II	66 (17.8)	180 (17.0)	
III	40 (10.8)	148 (14.0)	
IV	100 (27.0)	261 (24.6)	
**Surgery**			0.896
No	111 (29.9)	322 (30.4)	
Yes	260 (70.1)	737 (69.6)	
**Receiving chemotherapy or/and radiotherapy**			0.643
No	247 (66.6)	691 (65.3)	
Yes	124 (33.4)	368 (34.7)	
**OS**			
1	75.1 (70.6, 79.6)	65.9 (63.0, 68.8)	
3	56.0 (50.7, 61.3)	49.1 (46.0, 52.2)	
5	50.3 (44.8, 55.8)	43.0 (39.9, 46.1)	
**Median overall survival time**	62.0 (37.6, 86.4)	34.0 (26.1, 41.9)	
**CSS**			
1	77.7 (73.4, 82.0)	69.2 (66.5, 71.9)	
3	60.3 (55.0, 65.6)	54.2 (51.0, 57.3)	
5	57.4 (51.9, 62.9)	50.1 (47.0, 53.2)	
**Median cancer-specific survival**	NA	61.0 (34.3, 87.7)	

NEC: Neuroendocrine Carcinoma; AC: Adenocarcinoma; OS: Overall Survival; CSS: Cancer-specific Survival; IQR: Interquartile Range; NA: Not Available.

**Table 3:** Univariable and Multivariable Cox Regression Analyses of Factors Associated with Overall Survival of White Patients with Gastric Neuroendocrine Carcinoma and Adenocarcinoma in the Matched Data.

Clinicopathological Features	Univariable Analysis		Multivariable Analysis	
	HR (95% CI)	*p*	HR (95% CI)	*p*
**Age**	1.026 (1.020, 1.032)	<0.001	1.024 (1.018, 1.030)	<0.001
**Sex**				
Men	1[Reference]		1[Reference]	
Women	0.790 (0.688, 0.906)	0.001	0.937 (0.812, 1.080)	0.369
**Tumor location**				
Proximal	1[Reference]		1[Reference]	
Middle	0.818 (0.686, 0.976)	0.025	0.864 (0.719, 1.037)	0.116
Distal	0.844 (0.692, 1.030)	0.095	1.098 (0.891, 1.352)	0.381
Mix	1.497 (1.104, 2.209)	0.009	1.252 (0.916, 1.712)	0.158
Unknown	0.849 (0.648, 1.113)	0.235	0.979 (0.741, 1.293)	0.882
**Tumor size**	1.004 (1.003, 1.004)	<0.001	1.002 (1.001, 1.004)	<0.001
**T stage**			NA	NA
1	1[Reference]			
2	1.583 (1.270, 1.973)	<0.001		
3	2.548 (2.138, 3.036)	<0.001		
4a	3.710 (2.973, 4.629)	<0.001		
4b	5.417 (4.280, 6.857)	<0.001		
**N stage**			NA	NA
0	1[Reference]			
1	3.205 (2.737, 3.753)	<0.001		
2	2.740 (2.221, 3.381)	<0.001		
3a	3.591 (2.760, 4.673)	<0.001		
3b	6.650 (3.881, 11.394)	<0.001		
**M stage**			NA	NA
0	1[Reference]			
1	4.673 (4.037, 5.408)	<0.001		
**TNM stage**				
I	1[Reference]		1[Reference]	
II	2.711 (2.196, 3.346)	<0.001	2.645 (2.128, 3.287)	<0.001
III	4.603 (3.721, 5.694)	<0.001	4.669 (3.667, 5.946)	<0.001
IV	8.620 (7.164, 10.371)	<0.001	5.907 (4.721, 7.391)	<0.001
**Surgery**				
No	1[Reference]		1[Reference]	
Yes	0.239 (0.207, 0.275)	<0.001	0.387 (0.323, 0.464)	<0.001
**Receiving chemotherapy or/and radiotherapy**				
No	1[Reference]		1[Reference]	
Yes	2.718 (2.365, 3.123)	<0.001	0.772 (0.649, 0.920)	0.004
**Type**				
AC	1[Reference]		1[Reference]	
NEC	0.808 (0.685, 0.952)	0.011	0.719 (0.607, 0.853)	<0.001

NEC: Neuroendocrine Carcinoma; AC: Adenocarcinoma; OS: Overall Survival; CSS: Cancer-specific Survival; NA: Not Available.

**Table 4:** Univariable and Multivariable Cox Regression Analyses of Factors Associated with Cancer-specific Survival of White Patients with Gastric Neuroendocrine Carcinoma and Adenocarcinoma in the Matched Data.

Clinicopathological Features	Univariable Analysis		Multivariable Analysis	
	HR (95% CI)	*p*	HR (95% CI)	*p*
**Age**	1.017 (1.010, 1.024)	<0.001	1.016 (1.010, 1.023)	<0.001
**Sex**				
Men	1[Reference]		1[Reference]	
Women	0.756 (0.647, 0.883)	<0.001	0.951 (0.811, 1.116)	0.541
**Tumor location**				
Proximal	1[Reference]		1[Reference]	
Middle	0.771 (0.632, 0.941)	0.010	0.824 (0.670, 1.014)	0.067
Distal	0.766 (0.609, 0.962)	0.022	1.029 (0.810, 1.307)	0.814
Mix	1.539 (1.110, 2.135)	0.010	1.192 (0.851, 1.668)	0.307
Unknown	0.841 (0.624, 1.134)	0.256	0.933 (0.686, 1.268)	0.657
**Tumor size**	1.004 (1.003, 1.004)	<0.001	1.002 (1.001, 1.004)	<0.001
**T stage**			NA	NA
1	1[Reference]			
2	1.629 (1.254, 2.115)	<0.001		
3	3.060 (2.501, 3.743)	<0.001		
4a	4.562 (3.565, 5.837)	<0.001		
4b	7.046 (5.473, 9.071)	<0.001		
**N stage**			NA	NA
0	1[Reference]			
1	4.059 (3.398, 4.850)	<0.001		
2	3.161 (2.493, 4.008)	<0.001		
3a	4.471 (3.362, 5.944)	<0.001		
3b	8.739 (5.076, 15.046)	<0.001		
**M stage**			NA	NA
0	1[Reference]			
1	6.032 (5.144, 7.074)	<0.001		
**TNM stage**				
I	1[Reference]		1[Reference]	
II	4.177 (3.200, 5.453)	<0.001	4.091 (3.111, 5.380)	<0.001
III	7.424 (5.699, 9.673)	<0.001	7.624 (5.682, 10.231)	<0.001
IV	15.343 (12.136, 19.396)	<0.001	10.533 (8.004, 13.915)	<0.001
**Surgery**				
No	1[Reference]		1[Reference]	
Yes	0.201 (0.171, 0.235)	<0.001	0.377 (0.309, 0.461)	<0.001
**Receiving chemotherapy or/and radiotherapy**				
No	1[Reference]		1[Reference]	
Yes	3.338 (2.855, 3.901)	<0.001	0.752 (0.621, 0.911)	0.004
**Type**				
AC	1[Reference]		1[Reference]	
NEC	0.792 (0.659, 0.952)	0.013	0.691 (0.571, 0.835)	<0.001

NEC: Neuroendocrine Carcinoma; AC: Adenocarcinoma; OS: Overall Survival; CSS: Cancer-specific Survival; NA: Not Available.

## Data Availability

All data here are publicly available in the SEER database (https://seer.cancer.gov).

## Supplementary Materials

The following supporting information can be downloaded at: https://www.mdpi.com/article/10.3390/curroncol29070387/s1, Supplementary Table S1. Baseline Clinicopathologic Characteristics of White Patients with Early Stage Gastric Neuroendocrine Carcinoma and Adenocarcinoma in the Unmatched Data; Supplementary Table S2. Baseline Clinicopathologic Characteristics of White Patients with Early Stage Gastric Neuroendocrine Carcinoma and Adenocarcinoma in the Matched Data; Supplementary Table S3. Univariable and Multivariable Cox Regression Analyses of Factors Associated with Overall Survival of White Patients with Early Stage Gastric Neuroendocrine Carcinoma and Adenocarcinoma in the Matched Data; Supplementary Table S4. Univariable and Multivariable Cox Regression Analyses of Factors Associated with Cancer-specific Survival of White Patients with Early Stage Gastric Neuroendocrine Carcinoma and Adenocarcinoma in the Matched Data; Supplementary Table S5. Baseline Clinicopathologic Characteristics of White Patients with Locally Advanced Stage Gastric Neuroendocrine Carcinoma and Adenocarcinoma in the Unmatched Data; Supplementary Table S6. Baseline Clinicopathologic Characteristics of White Patients with Locally Advanced Stage Gastric Neuroendocrine Carcinoma and Adenocarcinoma in the Matched Data; Supplementary Table S7. Univariable and Multivariable Cox Regression Analyses of Factors Associated with Overall Survival of White Patients with Locally Advanced Stage Gastric Neuroendocrine Carcinoma and Adenocarcinoma in the Matched Data; Supplementary Table S8. Univariable and Multivariable Cox Regression Analyses of Factors Associated with Cancer-specific Survival of White Patients with Locally Advanced Stage Gastric Neuroendocrine Carcinoma and Adenocarcinoma in the Matched Data; Supplementary Table S9. Baseline Clinicopathologic Characteristics of White Patients with Distant Metastatic Stage Gastric Neuroendocrine Carcinoma and Adenocarcinoma in the Unmatched Data; Supplementary Table S10. Baseline Clinicopathologic Characteristics of White Patients with Distant Metastatic Stage Gastric Neuroendocrine Carcinoma and Adenocarcinoma in the Matched Data; Supplementary Table S11. Univariable and Multivariable Cox Regression Analyses of Factors Associated with Overall Survival of White Patients with Distant Metastatic Stage Gastric Neuroendocrine Carcinoma and Adenocarcinoma in the Matched Data; Supplementary Table S12. Univariable and Multivariable Cox Regression Analyses of Factors Associated with Cancer-specific Survival of White Patients with Distant Metastatic Stage Gastric Neuroendocrine Carcinoma and Adenocarcinoma in the Matched Data; Supplementary Table S13. Baseline Clinicopathologic Characteristics of East Asian Patients with Gastric Neuroendocrine Carcinoma and Adenocarcinoma in the Unmatched Data; Supplementary Table S14. Baseline Clinicopathologic Characteristic of East Asian Patients with Gastric Neuroendocrine Carcinoma and Adenocarcinoma in the Matched Data; Supplementary Figure S1. Schematic overview for patients identification; Supplementary Figure S2. Kaplan Meier Survival Curves for East Asian Patients with Gastric Neuroendocrine Carcinoma and Adenocarcinoma before (A: OS; B: CSS) and after PSM (C: OS; D: CSS).
