# Supplementary material for: Better Prognosis of Gastric Neuroendocrine Carcinoma Than Gastric Adenocarcinoma among Whites in the United States: A Propensity Score Matching Analysis Based on SEER
Source: Curr Oncol. 2022 Jul 11;29(7):4879–92. doi: 10.3390/curroncol29070387 (PMC9323980; doi:10.3390/curroncol29070387)
Supplement: Supplementary file 1 [file curroncol-29-00387-s001.zip › curroncol-1747370-supplementary.pdf]

SUPPLEMENT.

Supplementary Table S1. Baseline Clinicopathologic Characteristics of White Patients with Early Stage Gastric Neuroendocrine Carcinoma and Adenocarcinoma in the Unmatched Data

Supplementary Table S2. Baseline Clinicopathologic Characteristics of White Patients with Early Stage Gastric Neuroendocrine Carcinoma and Adenocarcinoma in the Matched Data

Supplementary Table S3. Univariable and Multivariable Cox Regression Analyses of Factors Associated with Overall Survival of White Patients with Early Stage Gastric Neuroendocrine Carcinoma and Adenocarcinoma in the Matched Data

Supplementary Table S4. Univariable and Multivariable Cox Regression Analyses of Factors Associated with Cancer-specific Survival of White Patients with Early Stage Gastric Neuroendocrine Carcinoma and Adenocarcinoma in the Matched Data

Supplementary Table S5. Baseline Clinicopathologic Characteristics of White Patients with Locally Advanced Stage Gastric Neuroendocrine Carcinoma and Adenocarcinoma in the Unmatched Data

Supplementary Table S6. Baseline Clinicopathologic Characteristics of White Patients with Locally Advanced Stage Gastric Neuroendocrine Carcinoma and Adenocarcinoma in the Matched Data

Supplementary Table S7. Univariable and Multivariable Cox Regression Analyses of Factors Associated with Overall Survival of White Patients with Locally Advanced Stage Gastric Neuroendocrine Carcinoma and Adenocarcinoma in the Matched Data

Supplementary Table S8. Univariable and Multivariable Cox Regression Analyses of Factors Associated with Cancer-specific Survival of White Patients with Locally Advanced Stage Gastric Neuroendocrine Carcinoma and Adenocarcinoma in the Matched Data

Supplementary Table S9. Baseline Clinicopathologic Characteristics of White Patients with Distant Metastatic Stage Gastric Neuroendocrine Carcinoma and Adenocarcinoma in the Unmatched Data

Supplementary Table S10. Baseline Clinicopathologic Characteristics of White Patients with Distant Metastatic Stage Gastric Neuroendocrine Carcinoma and Adenocarcinoma in the Matched Data

Supplementary Table S11. Univariable and Multivariable Cox Regression Analyses of Factors Associated with Overall Survival of White Patients with Distant Metastatic Stage Gastric Neuroendocrine Carcinoma and Adenocarcinoma in the Matched Data

Supplementary Table S12. Univariable and Multivariable Cox Regression Analyses of Factors Associated with Cancer-specific Survival of White Patients with Distant Metastatic Stage Gastric Neuroendocrine Carcinoma and Adenocarcinoma in the Matched Data

Supplementary Table S13. Baseline Clinicopathologic Characteristics of East Asian Patients with Gastric Neuroendocrine Carcinoma and Adenocarcinoma in the Unmatched Data

Supplementary Table S14. Baseline Clinicopathologic Characteristic of East Asian Patients with Gastric Neuroendocrine Carcinoma and Adenocarcinoma in the Matched Data

Supplementary Figure S1. Schematic overview for patients identification

Supplementary Figure S2. Kaplan Meier Survival Curves for East Asian Patients with Gastric Neuroendocrine Carcinoma and Adenocarcinoma before (A: OS; B: CSS) and after PSM (C: OS; D: CSS)

Supplementary Table S1. Baseline Clinicopathologic Characteristics of White Patients with Early Stage Gastric Neuroendocrine Carcinoma and Adenocarcinoma in the Unmatched Data

	No. (%) NEC (114)	No. (%) AC (2239)	P
**Characteristic**			
**Age, median (IQR)**	60.50 (50.75-70.25)	72 (62-80)	<0.001
**Sex**			<0.001
Men	39 (34.2)	1495 (66.8)	
Women	75 (65.8)	744 (33.2)	
**Tumor location**			<0.001
Proximal	14 (12.3)	1172 (52.3)	
Middle	61 (53.5)	452 (20.2)	
Distal	11 (9.6)	436 (19.5)	
Mix	1 (0.9)	65 (2.9)	
Unknown	27 (23.7)	114 (5.1)	
**Tumor size, median (IQR), cm**	0.5 (0.375-1.0)	2.0 (1.0-3.1)	<0.001
**Surgery**			0.179
No	17 (14.9)	449 (20.1)	
Yes	97 (85.1)	1790 (79.9)	
**Receiving chemotherapy or/and radiotherapy**			<0.001
No	112 (98.2)	1843 (82.3)	
Yes	2 (1.8)	396 (17.7)	

Supplementary Table S2. Baseline Clinicopathologic Characteristics of White Patients with Early Stage Gastric Neuroendocrine Carcinoma and Adenocarcinoma in the Matched Data

	No. (%) NEC (96)	No. (%) AC (263)	P
**Characteristic**			
**Age, median (IQR)**	62.50 (53.25-71)	63 (55-71)	0.426
**Sex**			0.566
Men	38 (39.6)	113 (43.0)	
Women	58 (60.4)	150 (57.0)	
**Tumor location**			<0.001
Proximal	10 (10.4)	151 (57.4)	
Middle	53 (55.2)	38 (14.4)	
Distal	7 (7.3)	55 (20.9)	
Mix	1 (1.0)	5 (1.9)	
Unknown	25 (26.1)	14 (5.4)	
**Tumor size, median (IQR), cm**	0.6 (0.4-1.0)	0.9 (0.4-1.5)	0.001
**Surgery**			0.674
No	9 (9.4)	21 (8.0)	
Yes	87 (90.6)	242 (92.0)	
**Receiving chemotherapy or/and radiotherapy**			1.000
No	94 (97.9)	257 (97.7)	
Yes	2 (2.1)	6 (2.3)	

Supplementary Table S3. Univariable and Multivariable Cox Regression Analyses of Factors Associated with Overall Survival of White Patients with Early Stage Gastric Neuroendocrine Carcinoma and Adenocarcinoma in the Matched Data

Clinicopathological features	Univariable analysis HR (95% CI)	P	Multivariable analysis HR (95% CI)	P
**Age**	1.069 (1.046, 1.092)	<0.001	1.062 (1.038, 1.087)	<0.001
**Sex**				
Men	1[Reference]		1[Reference]	
Women	0.687 (0.450, 1.049)	0.082	0.816 (0.515, 1.291)	0.384
**Tumor location**				
Proximal	1[Reference]		NA	NA
Middle	0.949 (0.551, 1.634)	0.851		
Distal	0.996 (0.559, 1.773)	0.988		
Mix	1.726 (0.416, 7.161)	0.452		
Unknown	0.755 (0.338, 1.686)	0.494		
**Tumor size**	1.007 (1.004, 1.010)	<0.001	1.007 (1.003, 1.011)	<0.001
**Surgery**				
No	1[Reference]		1[Reference]	
Yes	0.130 (0.078, 0.217)	<0.001	0.148 (0.079, 0.279)	<0.001
**Receiving chemotherapy or/and radiotherapy**				
No	1[Reference]		1[Reference]	
Yes	6.536 (2.996, 14.256)	<0.001	0.403 (0.151, 1.076)	0.070
**Type**				
AC	1[Reference]		1[Reference]	
GNEC	0.610 (0.337, 1.102)	0.102	0.528 (0.280, 0.996)	0.049

Supplementary Table S4. Univariable and Multivariable Cox Regression Analyses of Factors Associated with Cancer-specific Survival of White Patients with Early Stage Gastric Neuroendocrine Carcinoma and Adenocarcinoma in the Matched Data

Clinicopathological features	Univariable analysis HR (95% CI)	P	Multivariable analysis HR (95% CI)	P
**Age**	1.057 (1.024, 1.092)	0.001	1.044 (1.006, 1.083)	0.024
**Sex**				
Men	1[Reference]		1[Reference]	
Women	0.512 (0.267, 0.983)	0.044	0.705 (0.346, 1.437)	0.336
**Tumor location**				
Proximal	1[Reference]		NA	NA
Middle	0.549 (0.222, 1.355)	0.193		
Distal	0.713 (0.289, 1.759)	0.463		
Mix	1.587 (0.213, 11.840)	0.652		
Unknown	0.423 (0.099, 1.798)	0.244		
**Tumor size**	1.007 (1.003, 1.011)	0.002	1.008 (1.002, 1.015)	0.012
**Surgery**				
No	1[Reference]		1[Reference]	
Yes	0.085 (0.042, 0.171)	<0.001	0.098 (0.043, 0.225)	<0.001
**Receiving chemotherapy or/and radiotherapy**				
No	1[Reference]		1[Reference]	
Yes	7.632 (2.678, 21.751)	<0.001	0.382 (0.106, 1.377)	0.141
**Type**				
AC	1[Reference]		1[Reference]	
GNEC	0.390 (0.138, 1.104)	0.076	0.319 (0.105, 0.970)	0.044

Supplementary Table S5. Baseline Clinicopathologic Characteristics of White Patients with Locally Advanced Stage Gastric Neuroendocrine Carcinoma and Adenocarcinoma in the Unmatched Data

	No. (%) NEC (177)	No. (%) AC (8138)	P
**Characteristic**			
**Age, median (IQR)**	65 (53-73)	68 (59-77)	<0.001
**Sex**			0.002
Men	106 (59.9)	5757 (70.7)	
Women	71 (40.1)	2381 (29.3)	
**Tumor location**			<0.001
Proximal	65 (36.7)	4288 (52.7)	
Middle	54 (30.5)	1574 (19.3)	
Distal	24 (13.6)	1385 (17.0)	
Mix	11 (6.2)	472 (5.8)	
Unknown	23 (13.0)	419 (5.1)	
**Tumor size, median (IQR), cm**	3.0 (1.55-6.0)	3.0 (4.5-6.4)	<0.001
**T stage**			<0.001
1	17 (9.6)	502 (6.2)	
2	71 (40.1)	1465 (18.0)	
3	60 (33.9)	4139 (50.9)	
4a	20 (11.3)	1433 (17.6)	
4b	9 (5.1)	599 (7.4)	
**N stage**			<0.001
0	84 (47.5)	2357 (29.0)	
1	61 (34.5)	2839 (34.9)	
2	19 (10.7)	1568 (19.3)	
3a	10 (5.6)	1036 (12.7)	
3b	3 (1.7)	338 (4.2)	
**TNM stage**			<0.001
Ib	69 (39.0)	1159 (14.2)	
II	68 (38.4)	3540 (43.5)	
III	40 (22.6)	3439 (42.3)	
**Surgery**			0.667
No	34 (19.2)	1461 (18.0)	
Yes	143 (80.8)	6677 (82.0)	
**Receiving chemotherapy or/and radiotherapy**			<0.001
No	115 (65.0)	2605 (32.0)	
Yes	62 (35.0)	5533 (68.0)	

Supplementary Table S6. Baseline Clinicopathologic Characteristics of White Patients with Locally Advanced Stage Gastric Neuroendocrine Carcinoma and Adenocarcinoma in the Matched Data

	No. (%) NEC (159)	No. (%) AC (428)	P
**Characteristic**			
**Age, median (IQR)**	66 (55-75)	67 (58-75)	0.174
**Sex**			0.384
Men	99 (62.3)	283 (66.1)	
Women	60 (37.7)	145 (33.9)	
**Tumor location**			<0.001
Proximal	60 (37.7)	242 (56.5)	
Middle	46 (28.9)	90 (21.0)	
Distal	22 (13.8)	66 (15.4)	
Mix	11 (6.9)	13 (3.0)	
Unknown	20 (12.6)	17 (4.1)	
**Tumor size, median (IQR), cm**	3.5 (1.8-6.0)	2.5 (2.0-3.6)	<0.001
**T stage**			0.736
1	15 (9.4)	39 (9.1)	
2	58 (36.5)	138 (32.2)	
3	57 (35.8)	175 (40.9)	
4a	20 (12.6)	58 (13.6)	
4b	9 (5.7)	18 (4.2)	
**N stage**			0.692
0	70 (44.0)	195 (45.6)	
1	57 (35.8)	132 (30.8)	
2	19 (11.9)	67 (15.7)	
3a	10 (6.3)	28 (6.5)	
3b	3 (1.9)	6 (1.4)	
**TNM Stage**			0.640
Ib	54 (34.0)	128 (29.9)	
II	65 (40.9)	185 (43.2)	
III	40 (25.1)	115 (26.9)	
**Surgery**			0.513
No	26 (16.4)	80 (18.7)	
Yes	133 (83.6)	348 (81.3)	
**Receiving chemotherapy or/and radiotherapy**			0.471
No	97 (61.0)	247 (57.7)	
Yes	62 (39.0)	181 (42.3)	

Supplementary Table S7. Univariable and Multivariable Cox Regression Analyses of Factors Associated with Overall Survival of White Patients with Locally Advanced Stage Gastric Neuroendocrine Carcinoma and Adenocarcinoma in the Matched Data

Clinicopathological features	Univariable analysis HR (95% CI)	P	Multivariable analysis HR (95% CI)	P
**Age**	1.027 (1.018, 1.037)	<0.001	1.027 (1.018, 1.036)	<0.001
**Sex**				
Men	1[Reference]		1[Reference]	
Women	0.835 (0.675, 1.033)	0.097	0.833 (0.665, 1.044)	0.113
**Tumor location**				
Proximal	1[Reference]		1[Reference]	
Middle	0.647 (0.497, 0.843)	0.001	0.758 (0.572, 1.004)	0.054
Distal	0.852 (0.640, 1.135)	0.273	0.992 (0.728, 1.351)	0.959
Mix	0.785 (0.465, 1.325)	0.364	0.854 (0.496, 1.472)	0.570
Unknown	0.594 (0.371, 0.949)	0.030	0.769 (0.471, 1.254)	0.292
**Tumor size**	1.002 (1.001, 1.003)	0.002	1.002 (1.001, 1.003)	0.006
**T stage**			NA	NA
1	1[Reference]			
2	0.810 (0.549, 1.194)	0.286		
3	1.273 (0.879, 1.845)	0.202		
4a	1.993 (1.315, 3.020)	0.001		
4b	2.697 (1.594, 4.562)	<0.001		
**N stage**			NA	NA
0	1[Reference]			
1	1.501 (1.184, 1.902)	0.001		
2	1.678 (1.256, 2.241)	<0.001		
3a	2.481 (1.704, 3.611)	<0.001		
3b	4.558 (2.223, 9.347)	<0.001		
**TNM stage**				
Ib	1[Reference]		1[Reference]	
II	1.519 (1.176, 1.962)	0.001	1.529 (1.179, 1.983)	0.001
III	2.597 (1.985, 3.396)	<0.001	2.702 (2.006, 3.639)	<0.001
**Surgery**				
No	1[Reference]		1[Reference]	
Yes	0.360 (0.284, 0.457)	<0.001	0.375 (0.282, 0.498)	<0.001
**Receiving chemotherapy or/and radiotherapy**				
No	1[Reference]		1[Reference]	
Yes	1.716 (1.403, 2.099)	<0.001	0.900 (0.694, 1.168)	0.429
**Type**				
AC	1[Reference]		1[Reference]	
GNEC	0.725 (0.570, 0.923)	0.009	0.803 (0.621, 1.038)	0.094

Supplementary Table S8. Univariable and Multivariable Cox Regression Analyses of Factors Associated with Cancer-specific Survival of White Patients with Locally Advanced Stage Gastric Neuroendocrine Carcinoma and Adenocarcinoma in the Matched Data

Clinicopathological features	Univariable analysis HR (95% CI)	P	Multivariable analysis HR (95% CI)	P
**Age**	1.019 (1.009, 1.030)	<0.001	1.019 (1.009, 1.029)	<0.001
**Sex**				
Men	1[Reference]		1[Reference]	
Women	0.811 (0.633, 1.039)	0.097	0.863 (0.664, 1.121)	0.269
**Tumor location**				
Proximal	1[Reference]		1[Reference]	
Middle	0.630 (0.465, 0.855)	0.003	0.795 (0.574, 1.100)	0.166
Distal	0.772 (0.550, 1.083)	0.133	0.976 (0.679, 1.403)	0.896
Mix	0.816 (0.454, 1.466)	0.496	0.958 (0.520, 1.766)	0.891
Unknown	0.480 (0.267, 0.863)	0.014	0.710 (0.386, 1.307)	0.272
**Tumor size**	1.002 (1.001, 1.004)	0.001	1.002 (1.001, 1.004)	0.004
**T stage**			NA	NA
1	1[Reference]			
2	0.794 (0.490, 1.285)	0.347		
3	1.493 (0.948, 2.350)	0.083		
4a	2.347 (1.425, 3.864)	0.001		
4b	3.615 (2.011, 6.499)	<0.001		
**N stage**			NA	NA
0	1[Reference]			
1	1.718 (1.305, 2.261)	<0.001		
2	1.751 (1.245, 2.464)	0.001		
3a	2.854 (1.874, 4.348)	<0.001		
3b	6.100 (2.949, 12.619)	<0.001		
**Surgery**				
No	1[Reference]		1[Reference]	
Yes	0.331 (0.253, 0.432)	<0.001	0.371 (0.271, 0.508)	<0.001
**Receiving chemotherapy or/and radiotherapy**				
No	1[Reference]		1[Reference]	
Yes	2.094 (1.658, 2.644)	<0.001	1.043 (0.778, 1.397)	0.779
**Type**				
AC	1[Reference]		1[Reference]	
GNEC	0.675 (0.509, 0.896)	0.006	0.725 (0.535, 0.981)	0.037

Supplementary Table S9. Baseline Clinicopathologic Characteristics of White Patients with Distant Metastatic Stage Gastric Neuroendocrine Carcinoma and Adenocarcinoma in the Unmatched Data

	No. (%) NEC (101)	No. (%) AC (2458)	P
**Characteristic**			
**Age, median (IQR)**	65 (51-73)	65 (56-74)	0.255
**Sex**			<0.001
Men	58 (57.4)	1800 (73.2)	
Women	43 (42.6)	658 (26.8)	
**Tumor location**			0.147
Proximal	44 (43.6)	1342 (54.6)	
Middle	23 (22.8)	433 (17.6)	
Distal	17 (16.8)	320 (13.0)	
Mix	6 (5.9)	185 (7.5)	
Unknown	11 (10.9)	178 (7.2)	
**Tumor size, median (IQR), cm**	5.0 (3.0-7.3)	5.0 (3.5-7.5)	0.286
**T stage**			<0.001
1	16 (15.8)	651 (26.5)	
2	25 (24.8)	161 (6.6)	
3	22 (21.8)	719 (29.3)	
4a	12 (11.9)	390 (15.9)	
4b	26 (25.7)	537 (21.8)	
**N stage**			0.085
0	41 (40.6)	685 (27.9)	
1	44 (43.6)	1189 (48.4)	
2	9 (8.9)	340 (13.8)	
3a	6 (5.9)	214 (8.7)	
3b	1 (1.0)	30 (1.2)	
**Surgery**			0.639
No	67 (66.3)	1685 (68.6)	
Yes	34 (33.7)	773 (31.4)	
**Receiving chemotherapy or/and radiotherapy**			0.002
No	41 (40.6)	649 (26.4)	
Yes	60 (59.4)	1809 (73.6)	

Supplementary Table S10. Baseline Clinicopathologic Characteristics of White Patients with Distant Metastatic Stage Gastric Neuroendocrine Carcinoma and Adenocarcinoma in the Matched Data

	**No. (%)**<br>**NEC (99)**	**No. (%)**<br>**AC (277)**	**P**
**Characteristic**			
**Age, median (IQR)**	65 (51-73)	67 (54-73)	0.710
**Sex**			0.539
Men	58 (58.6)	172 (62.1)	
Women	41 (41.4)	105 (37.9)	
**Tumor location**			0.789
Proximal	43 (43.4)	132 (47.7)	
Middle	23 (23.2)	58 (20.9)	
Distal	16 (16.2)	38 (13.7)	
Mix	6 (6.1)	24 (8.7)	
Unknown	11 (11.1)	25 (9.0)	
**Tumor size, median (IQR), cm**	5.0 (3.0-7.3)	5.0 (3.0-6.8)	0.350
**T stage**			<0.001
1	16 (16.2)	91 (32.9)	
2	23 (23.2)	13 (4.7)	
3	22 (22.2)	75 (27.1)	
4a	12 (12.1)	43 (15.5)	
4b	26 (26.3)	55 (19.9)	
**N stage**			0.476
0	40 (40.4)	85 (30.7)	
1	43 (43.4)	131 (47.3)	
2	9 (9.1)	35 (12.6)	
3a	6 (6.1)	19 (6.9)	
3b	1 (1.0)	7 (2.5)	
**Surgery**			0.724
No	66 (66.7)	190 (68.6)	
Yes	33 (33.3)	87 (31.4)	
**Receiving chemotherapy or/and radiotherapy**			0.708
No	40 (40.4)	106 (38.3)	
Yes	59 (59.6)	171 (61.7)	

Supplementary Table S11. Univariable and Multivariable Cox Regression Analyses of Factors Associated with Overall Survival of White Patients with Distant Metastatic Stage Gastric Neuroendocrine Carcinoma and Adenocarcinoma in the Matched Data

Clinicopathological features	Univariable analysis HR (95% CI)	P	Multivariable analysis HR (95% CI)	P
**Age**	1.012 (1.004, 1.020)	0.003	1.009 (1.001, 1.017)	0.026
**Sex**				
Men	1[Reference]		1[Reference]	
Women	0.891 (0.717, 1.108)	0.299	0.912 (0.730, 1.140)	0.419
**Tumor location**				
Proximal	1[Reference]		1[Reference]	
Middle	0.990 (0.750, 1.305)	0.941	1.051 (0.789, 1.399)	0.735
Distal	1.023 (0.745, 1.406)	0.888	1.393 (0.998, 1.943)	0.051
Mix	1.054 (0.701, 1.586)	0.800	1.396 (0.896, 2.177)	0.140
Unknown	0.829 (0.564, 1.219)	0.341	0.968 (0.652, 1.438)	0.873
**Tumor size**	1.000 (0.999, 1.002)	0.789	1.000 (0.999, 1.002)	0.609
**T stage**			NA	NA
1	1[Reference]			
2	0.747 (0.503, 1.109)	0.148		
3	0.769 (0.575, 1.028)	0.077		
4a	1.033 (0.735, 1.452)	0.853		
4b	1.050 (0.779, 1.415)	0.751		
**N stage**			NA	NA
0	1[Reference]			
1	1.333 (1.043, 1.704)	0.022		
2	1.028 (0.718, 1.471)	0.880		
3a	1.248 (0.787, 1.977)	0.346		
3b	2.419 (1.172, 4.993)	0.017		
**Surgery**				
No	1[Reference]		1[Reference]	
Yes	0.648 (0.513, 0.820)	<0.001	0.450 (0.343, 0.590)	<0.001
**Receiving chemotherapy or/and radiotherapy**				
No	1[Reference]		1[Reference]	
Yes	0.640 (0.513, 0.797)	<0.001	0.463 (0.361, 0.594)	<0.001
**Type**				
AC	1[Reference]		1[Reference]	
GNEC	0.696 (0.543, 0.892)	0.004	0.655 (0.508, 0.843)	0.001

Supplementary Table S12. Univariable and Multivariable Cox Regression Analyses of Factors Associated with Cancer-specific Survival of White Patients with Distant Metastatic Stage Gastric Neuroendocrine Carcinoma and Adenocarcinoma in the Matched Data

Clinicopathological	Univariable analysis		Multivariable analysis	
features	HR (95% CI)	P	HR (95% CI)	P
**Age**	1.010 (1.002, 1.018)	0.012	1.007 (0.999, 1.015)	0.069
**Sex**				
Men	1[Reference]		1[Reference]	
Women	0.903 (0.724, 1.127)	0.368	0.934 (0.745, 1.171)	0.555
**Tumor location**				
Proximal	1[Reference]		1[Reference]	
Middle	0.982 (0.741, 1.301)	0.899	1.054 (0.788, 1.409)	0.725
Distal	0.961 (0.692, 1.335)	0.813	1.322 (0.937, 1.865)	0.111
Mix	0.991 (0.649, 1.512)	0.966	1.314 (0.830, 2.079)	0.244
Unknown	0.854 (0.581, 1.257)	0.424	1.005 (0.677, 1.493)	0.980
**Tumor size**	1.000 (0.999, 1.002)	0.811	1.000 (0.999, 1.002)	0.649
**T stage**			NA	NA
1	1[Reference]			
2	0.704 (0.466, 1.061)	0.094		
3	0.752 (0.559, 1.014)	0.061		
4a	1.075 (0.763, 1.515)	0.680		
4b	1.074 (0.793, 1.453)	0.645		
**N stage**			NA	NA
0	1[Reference]			
1	1.409 (1.095, 1.814)	0.008		
2	1.077 (0.747, 1.553)	0.690		
3a	1.274 (0.794, 2.044)	0.315		
3b	2.591 (1.253, 5.358)	0.010		
**Surgery**				
No	1[Reference]		1[Reference]	
Yes	0.616 (0.483, 0.784)	<0.001	0.435 (0.329, 0.575)	<0.001
**Receiving chemotherapy or/and radiotherapy**				
No	1[Reference]		1[Reference]	
Yes	0.665 (0.531, 0.834)	<0.001	0.473 (0.367, 0.610)	<0.001
**Type**				
AC	1[Reference]		1[Reference]	
GNEC	0.689 (0.535, 0.888)	0.004	0.649 (0.501, 0.840)	0.001

Supplementary Table S13. Baseline Clinicopathologic Characteristics of East Asian Patients with Gastric Neuroendocrine Carcinoma and Adenocarcinoma in the Unmatched Data

	No. (%) NEC (21)	No. (%) AC (2092)	P
**Characteristic**			
**Age, median (IQR)**	69 (60-79)	72 (62-79)	0.469
**Sex**			0.739
Men	14 (66.7)	1321 (63.1)	
Women	7 (33.3)	771 (36.9)	
**Tumor location**			0.135
Proximal	7 (33.3)	294 (14.1)	
Middle	4 (19.0)	680 (32.5)	
Distal	7 (33.3)	810 (38.7)	
Mix	1 (4.8)	165 (7.9)	
Unknown	2 (9.5)	143 (6.8)	
**Tumor size, median (IQR), cm**	4.6 (2.8-8.85)	4.0 (2.2-6.1)	0.185
**T stage**			0.797
1	6 (28.6)	666 (31.8)	
2	4 (19.0)	232 (11.1)	
3	6 (28.6)	641 (30.6)	
4a	3 (14.3)	368 (17.6)	
4b	2 (9.5)	185 (8.8)	
**N stage**			0.447
0	11 (52.4)	932 (44.6)	
1	7 (33.3)	481 (23.0)	
2	2 (9.5)	319 (15.2)	
3a	1 (4.8)	249 (11.9)	
3b	0 (0.0)	111 (5.3)	
**M stage**			0.218
0	16 (76.2)	1788 (85.5)	
1	5 (23.8)	304 (14.5)	
**TNM stage**			0.464
I	6 (28.6)	706 (33.7)	
II	6 (28.6)	474 (22.7)	
III	4 (19.0)	608 (29.1)	
IV	5 (23.8)	304 (14.5)	
**Surgery**			0.045
No	6 (28.6)	268 (12.8)	
Yes	15 (71.4)	1824 (87.2)	
**Receiving chemotherapy or/and radiotherapy**			0.128
No	15 (71.4)	1135 (54.3)	
Yes	6 (28.6)	957 (45.7)	

Supplementary Table S14. Baseline Clinicopathologic Characteristic of East Asian Patients with Gastric Neuroendocrine Carcinoma and Adenocarcinoma in the Matched Data

	No. (%)<br>NEC (18)	No. (%)<br>AC (48)	P
**Characteristic**			
**Age, median (IQR)**	69.5 (64-80.25)	71 (62-84)	0.948
**Sex**			0.319
Men	13 (72.2)	40 (83.3)	
Women	5 (27.8)	8 (16.7)	
**Tumor location**			0.142
Proximal	6 (33.3)	4 (8.3)	
Middle	4 (22.2)	20 (41.7)	
Distal	6 (33.3)	17 (35.4)	
Mix	1 (5.6)	4 (8.3)	
Unknown	1 (5.6)	3 (6.3)	
**Tumor size, median (IQR), cm**	5.250 (2.825-8.775)	2.5 (1.5-6.025)	0.044
**T stage**			0.906
1	5 (27.8)	14 (29.2)	
2	4 (22.2)	6 (12.5)	
3	5 (27.8)	16 (33.3)	
4a	2 (11.1)	7 (14.6)	
4b	2 (11.1)	5 (10.4)	
**N stage**			0.792
0	9 (50.0)	22 (45.8)	
1	6 (33.3)	12 (25.0)	
2	2 (11.1)	5 (10.4)	
3a	1 (5.6)	8 (16.7)	
3b	0 (0.0)	1 (2.1)	
**M stage**			0.739
0	14 (77.8)	39 (81.3)	
1	4 (22.2)	9 (18.8)	
**TNM stage**			0.981
I	5 (27.8)	13 (27.1)	
II	5 (27.8)	16 (33.3)	
III	4 (22.2)	10 (20.8)	
IV	4 (22.2)	9 (18.8)	
**Surgery**			0.751
No	5 (27.8)	11 (22.9)	
Yes	13 (72.2)	37 (77.1)	
**Receiving chemotherapy or/and radiotherapy**			0.778
No	12 (66.7)	29 (60.4)	
Yes	6 (33.3)	19 (39.6)	

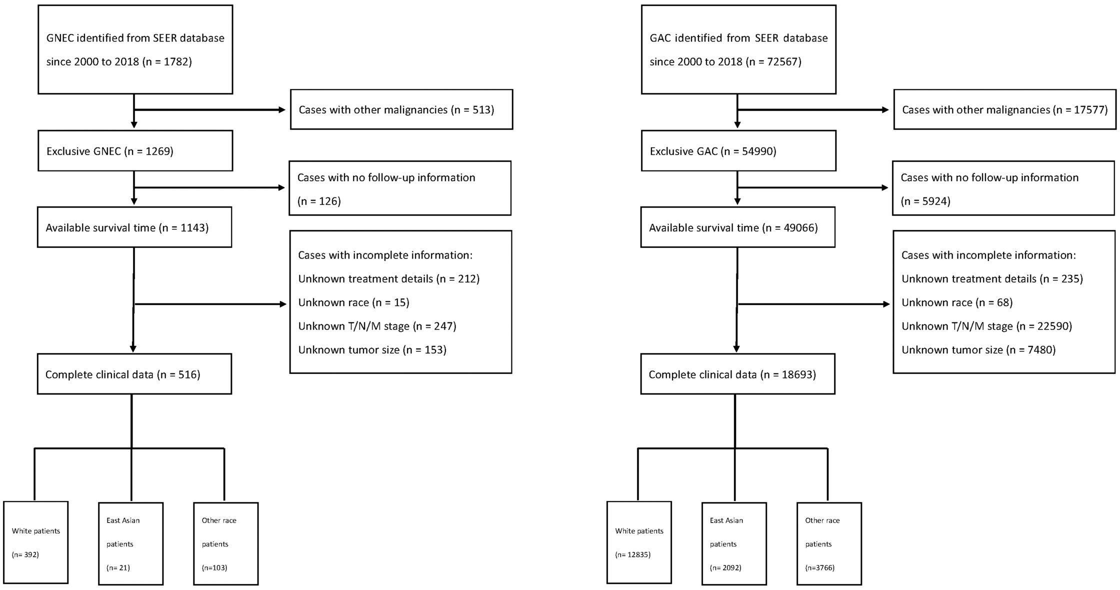

Supplementary Figure S1. Schematic overview for patients identification

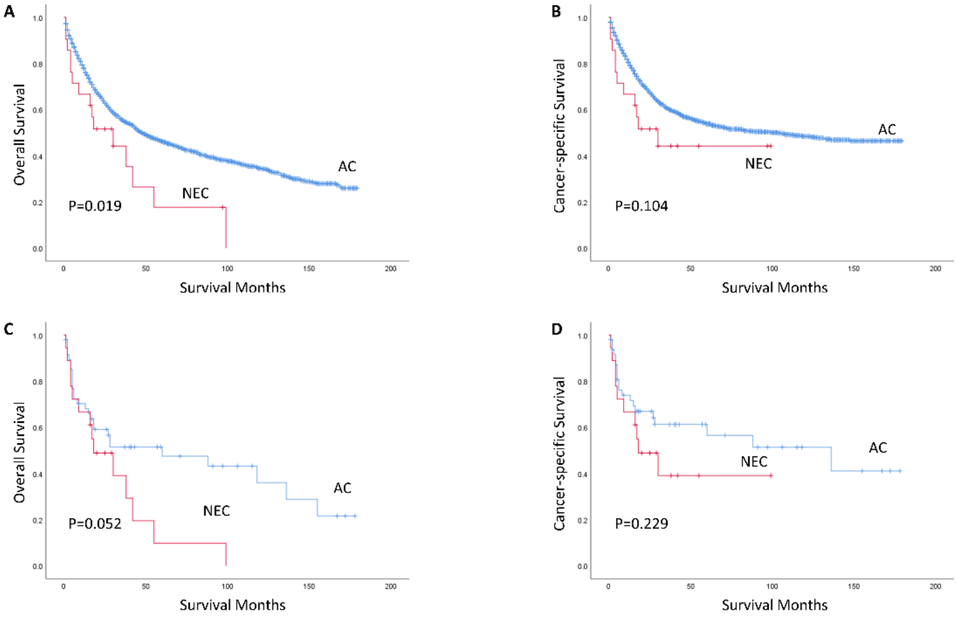

Supplementary Figure S2. Kaplan Meier Survival Curves for East Asian Patients with Gastric Neuroendocrine Carcinoma and Adenocarcinoma before (A: OS; B: CSS) and after PSM (C: OS; D: CSS)
